# Current epidemiological and experimental evidence relating the chemical exposome to Alzheimer’s disease

**DOI:** 10.1002/alz.087443

**Published:** 2025-01-09

**Authors:** Sophie Lefèvre‐Arbogast, Jade Chaker, Fabien Mercier, Robert Barouki, Xavier Coumoul, Gary W Miller, Arthur David, Cécilia Samieri

**Affiliations:** ^1^ Bordeaux Population Health Research Center, Inserm U1219, University of Bordeaux, Bordeaux France; ^2^ Univ Rennes, Inserm, EHESP, Irset (Institut de Recherche en Santé, Environnement et Travail), Rennes, NA France; ^3^ Université Paris Cité, T3S, INSERM UMR‐S 1124, Paris, NA France; ^4^ Mailman School of Public Health, Columbia University, New York, NY USA

## Abstract

This critical overview aims to provide one of the most comprehensive synthesis of epidemiological and experimental evidence relating the chemical exposome to Alzheimer’s Disease (AD). We have focused on chemical pollutants that mostly result from anthropic activities and involved in toxicology pathways, including pesticides, organic solvents, metals, combustion air pollutants, dioxins, flame retardants, fluorosurfactants, plastic components and food/cosmetic additives. In total, we have reviewed over 120 epidemiological studies examining the link between chemical exposures and the risk of AD or cognitive impairment in older adults, along with more than 250 experimental studies assessing the impact of chemical exposures on neurodegeneration‐related pathways.

The predominant emphasis in epidemiological research has been on pesticides, metals, solvents and combustion air pollutants. The most consistently observed associations with AD/cognitive impairment involve occupational exposure to pesticides and exposure to fine particulate matter. Less is known about the impact of lower levels of pesticide exposure in the general population, with emerging evidence supported by a few studies. Conversely, in the general population, there is growing evidence of the role of air pollution, especially increased exposure to fine particulate matters, in AD. Regarding metals and solvents, associations with AD have been suggested in several case‐control or cross‐sectional studies but the overall weight of evidence remains low. In experimental studies, pesticides and metals have also been the most extensively investigated and predominantly implicated in the disruption of neurotransmission, oxidative stress and mitochondrial dysfunction; some pesticides might also specifically target amyloid‐β aggregation.

Finally, our comprehensive literature review highlights a gap in knowledge concerning the influence of many chemical exposures on AD, especially the most recent pesticide families as well as synthetic additive chemicals considered as ubiquitous. Yet, some of these chemicals have been incriminated in neurodevelopmental defects, and there exists suggestive, albeit still limited, evidence of their involvement in neurodegeneration‐related pathways, such as neurotransmission disruption, supporting the need for further research efforts.

